# Newly found leaf arrangement to reduce self-shading within a crown in Japanese monoaxial tree species

**DOI:** 10.1007/s10265-024-01524-5

**Published:** 2024-01-28

**Authors:** Hitoshi Aoyagi, Miyabi Nakabayashi, Toshihiro Yamada

**Affiliations:** Graduate School of Integrated Sciences for Life, Hiroshima University, 1-7-1 Kagamiyama, Higashi-Hiroshima, Hiroshima 739-8521 Japan

**Keywords:** Araliaceae, Crown structure, Leaf arrangement, Petiole, Tree morphology, Self-shading

## Abstract

A newly found leaf arrangement to reduce self-shading was observed in a Japanese warm-temperate forest. For monoaxial trees that deploy leaves directly on a single stem, leaf arrangements involving progressive elongation of the petiole and progressive increase in deflection angle (the angle between stem and petiole) from the uppermost to the lowermost leaves act to reduce self-shading. However, the progressive reduction in petiole length and deflection angle from the uppermost to the lowermost leaves should also result in the reduction of self-shading. Nevertheless, the latter leaf arrangement has not been reported previously for any tree species. Four Araliaceae species, namely, *Gamblea innovans*, *Chengiopanax sciadophylloides*, *Dendropanax trifidus* and *Fatsia japonica*, which are typical monoaxial tree species in Japan, were studied. We examined the crown structure of saplings growing in the light-limited understorey in a Japanese warm-temperate forest. Two evergreen species, *Dendropanax trifidus* and *F. japonica* showed progressive petiole elongation and progressive increase in the deflection angle from the uppermost to the lowermost leaves. In contrast, saplings of deciduous species, *G. innovans* and *C. sciadophylloides* had a leaf arrangement involving progressive reduction in petiole length and deflection angle from the uppermost to the lowermost leaves. The leaf arrangement has diversified among members of the same family, but all four studied species develop a crown with little self-shading that is adapted for growth in the light-limited understorey. Although trees are likely to be under the same selective pressure to reduce self-shading, this study revealed that there is flexibility in its morphological realisation, which has been poorly appreciated previously.

## Introduction

In a forest with a closed canopy, the amount of light reaching the understorey is limited (Bovolenta et al. 2022; Chazdon and Fetcher 1984; Clark and Clark 1999; Poorter and Arets 2003; Yamada et al. 2014). Light penetration to the understorey is predominantly from the zenith (Horn 1971; Turton 1992). Therefore, trees in the understorey are required to efficiently capture the limited light from the zenith for photosynthesis. For these trees, reduction of self-shading, which is the shading of leaves within an individual tree, is of paramount importance because the photosynthetic rate is reduced in a shaded leaf. Subsequently, reduced photosynthesis due to shading will lead to a decline in crown-level productivity (Coops et al. 2017; Kitajima et al. 2005; Niinemets 2007).

Horn (1971) and Chazdon (1985) noted that, for trees in the forest understorey, the optimal arrangement of leaves for photosynthesis is in one layer at a given height without overlap of leaves within a crown. Trees often realise this leaf arrangement. For example, Yamada and Suzuki (1996) examined the crown structure of *Scaphium macropodum*, an evergreen tree species in Malvaceae in a Bornean rainforest. In this species, numerous large leaves are attached directly on a branchless stem (herein, we term this growth form “monoaxial”). This species reduces self-shading by adjusting the petiole length within a crown (Yamada and Suzuki 1996); the leaf at the top of the stem has the shortest petiole and the petiole is progressively longer toward the lowermost leaf. Monoaxial saplings of *Macaranga semiglobosa*, an evergreen tree species in Euphorbiaceae (Takahashi and Mikami 2008), *Macaranga rostulata*, an evergreen tree species in Euphorbiaceae, *Homalanthus caloneurus* (Miyazawa et al. 2006), an evergreen tree species in Euphorbiaceae (Miyazawa et al. 2006) and *Kalopanax pictus*, a deciduous tree species in Araliaceae (Seino 2001) realise the reduction of self-shading by adjusting the petiole length in the same manner as *S. macropodum*.

**Fig. 1:**
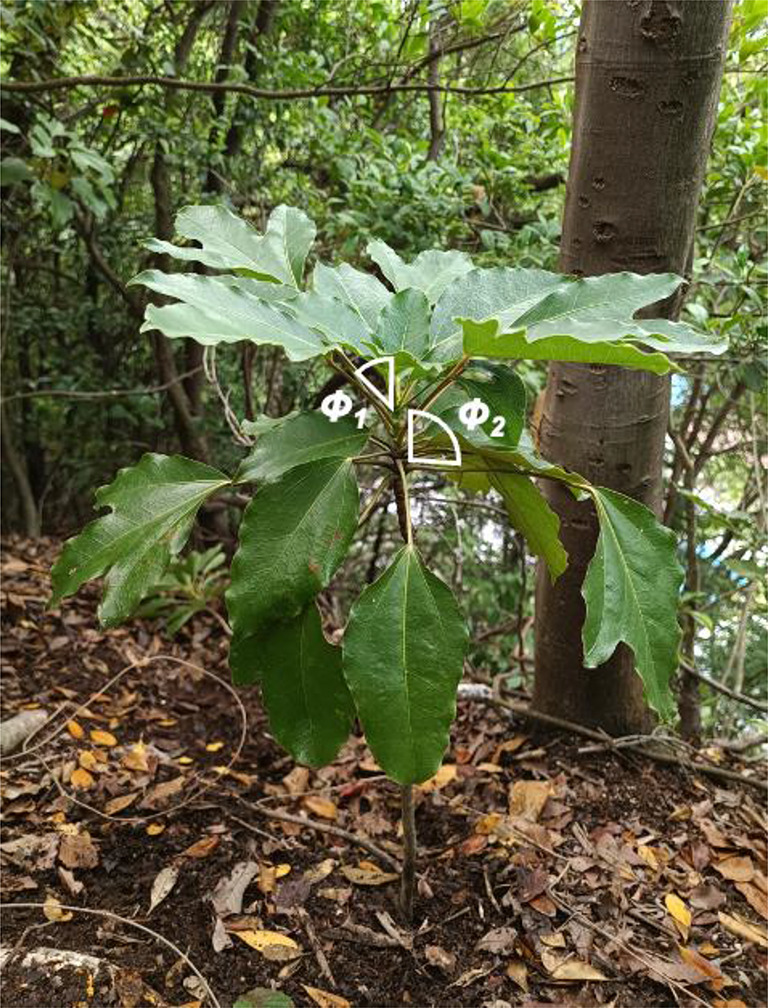
A sapling of *Dendropanax trifidus* showing deflection angle (angle between stem and leaf petiole, *Φ*). *Φ*_1_ shows example of a small deflection angle, while *Φ*_2_ show an example of a large deflection angle. The progressive increase in deflection angle from the top to the bottom work to reduce self-shading, because leaves are gradually located to further position from the stem from the top to the bottom leaves, even though petiole lengths are identical among the leaves

In addition to petiole length, the angle between the stem and petiole (hereafter, this is termed the “deflection angle”; 0° = a petiole oriented towards the zenith and 90° = a petiole oriented towards the horizon; Fig. 1) contributes to the reduction in self-shading for monoaxial trees (Kikuzawa et al. 1996). In the case of *S. macropodum*, the uppermost leaf is directed towards the zenith and the lowermost leaf is oriented horizontally (Yamada and Suzuki 1996).

Previous studies have commonly shown that adjustment of the petiole length (Brites and Valladares 2005; Furqoni et al. 2018; Takahashi and Mikami 2008; Takenaka 1994; Yamada and Suzuki 1996) and deflection angle (Kikuzawa et al. 1996; Niklas 1988; Yamada and Suzuki 1996) act to reduce self-shading. In these studies, only the progressive elongation of the petiole and the progressive increase in deflection angle from the uppermost to the lowermost leaves were reported. However, progressive reduction in the petiole length as well as progressive reduction in the deflection angle from the uppermost to the lowermost leaves can create a similar crown structure to that of the above-mentioned species and can reduce self-shading.

Abovementioned knowledge is about the leaf arrangement to reduce self-shading among leaves that appear simultaneously in a year (herein, we term the leaves that emerge in one flush a “leaf cluster”). For evergreen trees that retain each leaf for multiple years and have a crown which consists of leaf clusters that appeared in different years, self-shading between the leaf clusters (shading of the lower, older leaves by the newer leaves) (Takenaka 1997) is another problem to avoid. However, this self-shading does not occur for deciduous trees that drop their leaves every fall and have a crown which shows only the leaves developed in the year. Accordingly, between-cluster self-shading is specific to evergreen trees.

In a Japanese warm-temperate forest, we encountered certain monoaxial tree species in the Araliaceae that adopt progressive reduction in the petiole length or progressive reduction in the deflection angle from the uppermost to the lowermost leaves to avoid self-shading. Hence, we examined the crown structure of these tree species aiming to understand adaptive leaf arrangements to reduce self-shading in terms of within a leaf cluster and between leaf clusters.

## Materials and methods

### Species studied

We studied four species of Araliaceae in a Japanese warm-temperate forest (Table 1). These species had a monoaxial growth habit and the large leaves were attached directly to the stem without branches. The species studied were *Gamblea innovans* (Siebold & Zucc.) C.B.Shang, Lowry & Frodin, *Chengiopanax sciadophylloides* (Franch. & Sav.) C.B.Shang & J.Y.Huang, *Dendropanax trifidus* (Thunb.) Makino ex H.Hara, and *Fatsia japonica* (Thunb.) Decne. & Planch. A previous phylogenetic study using chromosomal DNA supported the segregation of the four genera (Yi et al. 2004), but the phylogenetic relationships among the genera have not been resolved.

**Table 1:** Tree species of Araliaceae examined in this study

Species	Tree size	Maximum attainable height	Leaf longevity	Leaf morphology
*Chengiopanax sciadophylloides*	Subcanopy	10–12 m	Deciduous	Palmately compound leaf
*Dendropanax trifidus*	Subcanopy	9–15 m	Evergreen	Trilobed palmately-incised simple leaf*
*Fatsia japonica*	Understorey	3–5 m	Evergreen	Palmately-incised simple leaf
*Gamblea innovans*	Subcanopy	15 m	Deciduous	Palmately compound leaf

*Occasionally bilobed palmately-incised leaf or simple leaf with entire margin

*G. innovans* is a subcanopy deciduous tree. Mature individuals reach 15 m in height (Table 1). This species has palmately compound leaves that are 7–30 cm in length from the petiole base to the leaf tip. Each leaf is composed of three leaflets. *C. sciadophylloides* is a subcanopy deciduous tree. Mature individuals are 10–12 m in height. The leaves of this species are palmately compound and 8–30 cm in length from the base of the petiole to the tip of the leaf. An individual leaf is composed of five leaflets. *D. trifidus* is a subcanopy evergreen tree. Mature individuals attain a height of 9–15 m. This species has trilobed palmately-incised leaves that are 5–12 cm in length. *F. japonica* is an understorey evergreen tree. Mature individuals are 3–5 m in height. It develops palmately-incised leaves that are 35–85 cm in length with five to seven lobes. All species are shade tolerant and grow under closed canopies.

All four species develop a number of leaves with spiral phyllotaxy once per year during a short period in spring (usually in April). The number of leaves in one cluster differs among the species. The deciduous species shed all leaves on an individual tree in late autumn (usually in November). In contrast, the leaves of the evergreen species remain functional for 3–5 years, and thus three to five leaf clusters can be present on a single tree. We defined each leaf cluster as ‘topmost’, ‘second’, ‘third’, etc., in order from the shoot apex.

### Study sites

Saplings of *G. innovans* and *C. sciadophylloides* growing in a broad-leaved evergreen forest on Mt. Gagara (34°24’N, 132°43’E), Higashi-Hiroshima, Hiroshima, Japan were studied. This is a secondary forest that regenerated after human disturbance, including timber harvest. The forest was dominated by evergreen species, such as *Quercus glauca*, *Ilex pedunculosa* and *Symplocos lucida*. The average forest canopy height was approximately 15 m. The altitude of the study area is between 225 and 330 m above sea level. At the Higashi-Hiroshima Meteorological Observatory, which is approximately 2700 m northwest of the study site, the annual mean temperature and mean annual precipitation between 2013 and 2022 were 14.0 °C and 1564 mm, respectively.

Saplings of *D. trifidus* and *F. japonica* in a broad-leaved evergreen forest on Mt. Ushita (34°25’N, 132°29’E), Hiroshima, Japan were examined because these species were uncommon in the forest on Mt. Gagara. The study area consisted of secondary forests affected by wildfire and timber harvest, which were dominated by evergreen species, such as *Lithocarpus glaber* and *Pinus densiflora*. The average forest canopy height was approximately 15 m. The altitude of the study area is between 134 and 260 m above sea level. The annual mean temperature between 2013 and 2022 at the Hiroshima Meteorological Weather Station, which is located approximately 1160 m southwest of the study site, was 16.8 °C. The mean annual precipitation was 1754 mm for the same 10-year period.

### Field methods

Data collection was conducted between May and July 2022. Because we were interested in whether the crown structure reduces self-shading in the understorey, we sampled trees growing under a closed canopy. No scars or breakage and little deviation of the stem from vertical were noted for all sampled trees.

Ten saplings ranging from 95 to 278 cm in height were sampled for each species.

The height of the sampled trees was measured. For all leaves on the sampled trees, we measured the petiole length and the deflection angle (*Φ*) between the stem and petiole using protractor (0° = petiole oriented toward the zenith and 90° = petiole oriented toward the horizon; Fig. 1). We numbered the node for each individual leaf according to the position of the leaf within a crown; the topmost leaf was designated node 1, the second leaf from the top was node 2, and so forth.

### Data analysis

We calculated the Spearman’s rank correlation coefficient (Spearman’s *ρ*) between the node order and petiole length. If a tree shows progressive elongation of the petiole from the uppermost to the lowermost leaves, the correlation coefficient will be positive. In contrast, if a tree shows a progressive reduction in petiole length from the uppermost to the lowermost leaves, a negative correlation will be observed. We also analysed the correlation between node order and deflection angle. If a tree shows a progressive increase in deflection angle from the uppermost to the lowermost leaves, the correlation coefficient will be positive. However, if a tree shows a progressive reduction of the deflection angle from the uppermost to the lowermost leaves, a negative correlation will be observed. Finally, we calculated the mean Spearman’s *ρ* for each species. We then compared the means among the four species by performing an analysis of variance (ANOVA) followed by multiple comparisons using the Tukey–Kramer test after first confirming the assumptions of normality and homogeneity of variance. For this analysis, we focused only on leaves in the topmost leaf cluster (the leaf cohort that emerged in 2022).

As mentioned already, three to five leaf clusters may be present on an individual tree of the evergreen species (*D. trifidus* and *F. japonica*). To compare the petiole length and deflection angle between leaf clusters, we compared the mean petiole length and mean deflection angle between the leaf clusters by performing a repeated measures ANOVA followed by a paired-samples *t-*test after confirming the assumptions of normality and homogeneity of variance. The third cluster or older clusters (i.e., clusters of age 3 years and older) comprised few leaves, possibly because of the leaf longevity of the species studied. Therefore, for this analysis we compared petiole length and deflection angle between the topmost and the second leaf clusters only. All statistical analyses were performed with R version 4.2.2.

## Results

### Leaf arrangement within the topmost cluster

**Fig. 2:**
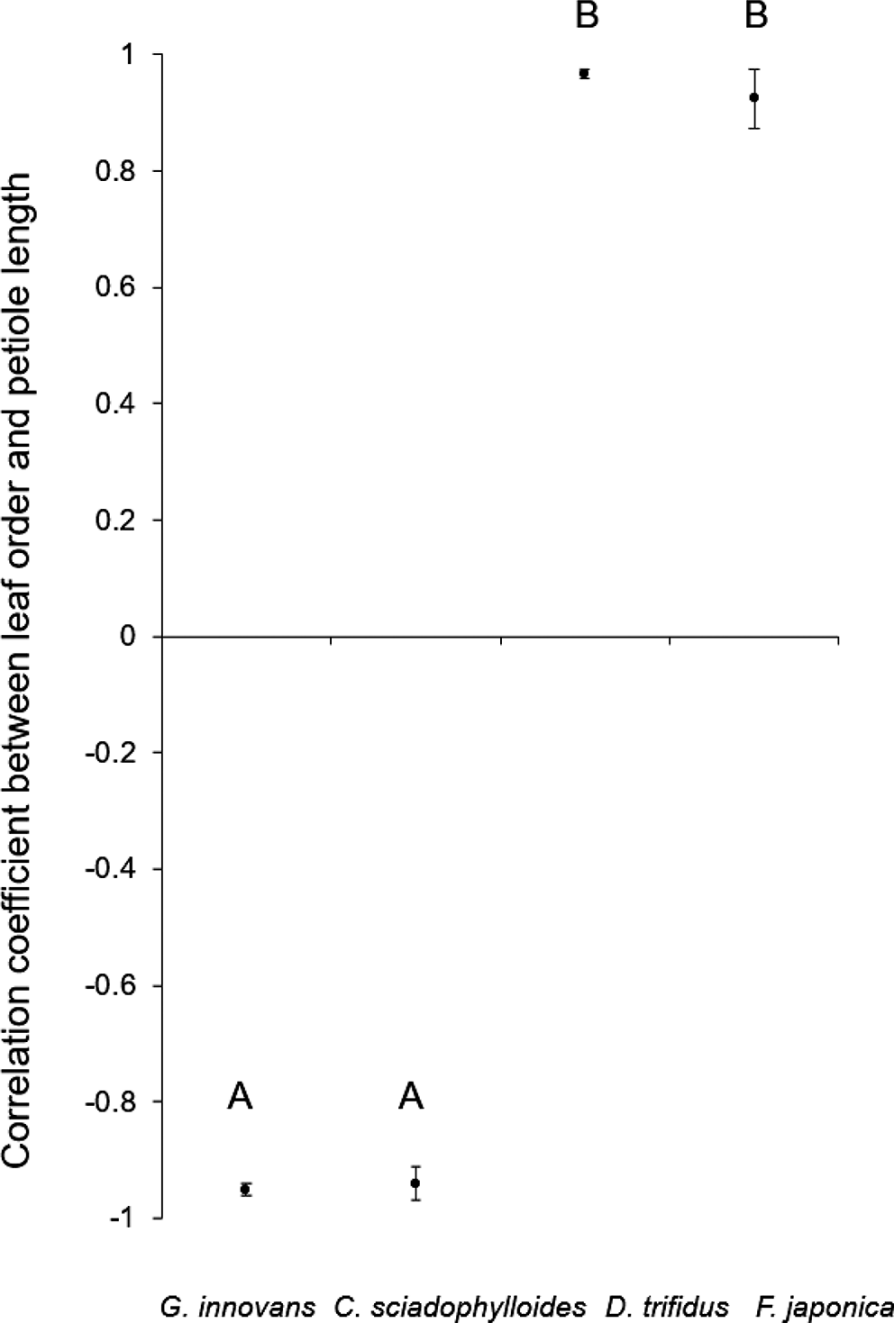
Mean Spearman’s *ρ* between node order and petiole length for *Gamblea innovans* (*n =* 10), *Chengiopanax sciadophylloides* (*n =* 10), *Dendropanax trifidus* (*n =* 10) and *Fatsia japonica* (*n =* 10). Error bars indicate the standard error for each species. The results of a Tukey–Kramer pairwise comparison are indicated by letters above each box; different letters represent a significant difference between the correlation coefficients (Tukey–Kramer, *p* < 0.01)

**Fig. 3:**
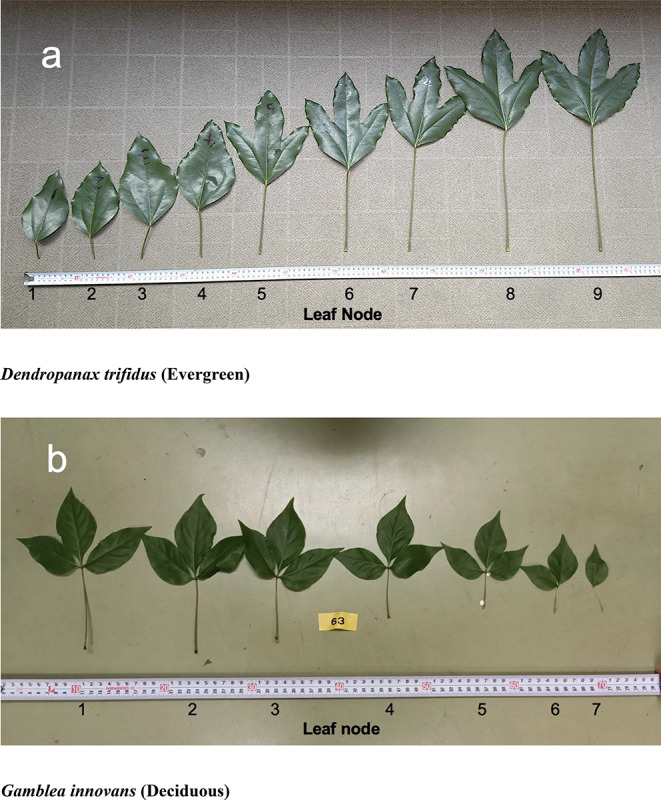
Leaves in the top cluster (2022 cohort) of *Dendropanax trifidus* (**a**, evergreen) and *Gamblea innovans* (**b**, deciduous). Numbers under each leaf indicate leaf order from the top in the cluster (The top leaf = 1, the bottom leaf = the largest figure)

The mean Spearman’s *ρ* between the node order and petiole length was strongly significantly positive for *D. trifidus* and *F. japonica*: evergreen species (0.97 and 0.92, respectively). In contrast, the values for *G. innovans* and *C. sciadophylloides* (deciduous species) were strongly negative (− 0.95 and − 0.94, respectively). The mean Spearman’s *ρ* differed significantly among the four species (ANOVA followed by the Tukey–Kramer test for multiple comparisons, *p* < 0.01; Fig. 2). These results revealed that the topmost leaf had the shortest petiole and the petiole was gradually elongated with increase in node order for *D. trifidus* and *F. japonica*, whereas the uppermost leaf of *G. innovans* and *C. sciadophylloides* had the longest petiole and the petiole was shortened with increase in node order (Fig. 3).

**Fig. 4:**
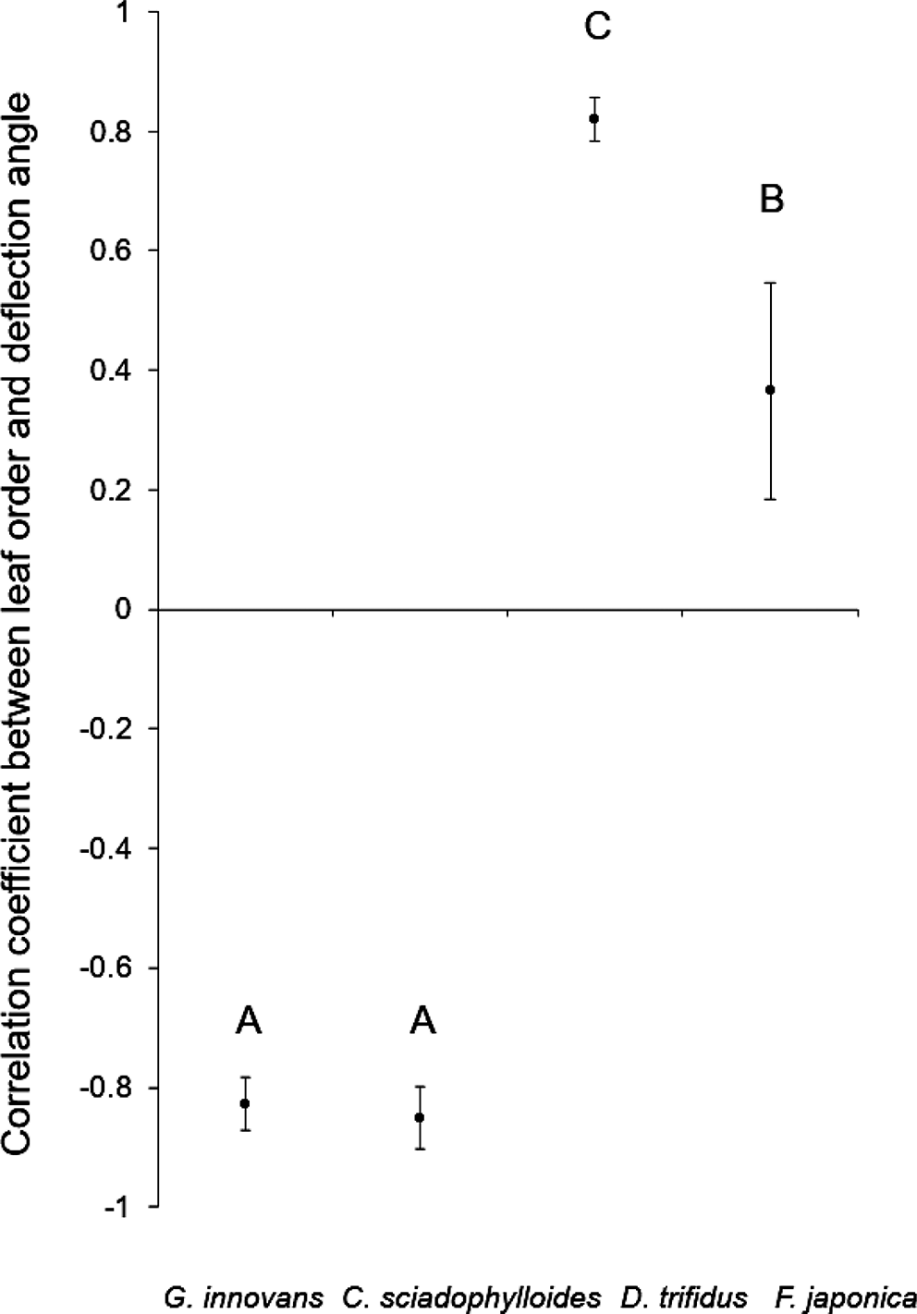
Mean Spearman’s *ρ* between node order and deflection angle for *Gamblea innovans* (*n =* 10), *Chengiopanax sciadophylloides* (*n =* 10), *Dendropanax trifidus* (*n =* 10) and *Fatsia japonica* (*n =* 10). Error bars represent the standard error for each species. The results of a Tukey**–**Kramer pairwise comparison are indicated by letters above each box; different uppercase letters represent a significant difference between the correlation coefficients (Tukey**–**Kramer, *p* < 0.01)

The deflection angle between the stem and petiole decreased with the increase in node order for *G. innovans* and *C. sciadophylloides*, in contrast to *D. trifidus* and *F. japonica*, for which the deflection angle increased as the node order increased. The mean Spearman’s *ρ* between the deflection angle and node order for *D. trifidus* and *F. japonica* was 0.82 and 0.37, respectively, and that for *G. innovans* and *C. sciadophylloides* was − 0.83 and − 0.85, respectively (Fig. 4). Significant differences in mean Spearman’s *ρ* among the four species were observed (ANOVA followed by the Tukey–Kramer test for multiple comparisons, *p* < 0.01). Therefore, for *D. trifidus* and *F. japonica*, the uppermost leaf was directed in the zenith and the underlying leaves were gradually tilted horizontally with increase in node order, whereas the uppermost leaf of *G. innovans* and *C. sciadophylloides* was oriented horizontally and the lowermost leaf was directed in the zenith.

**Fig. 5:**
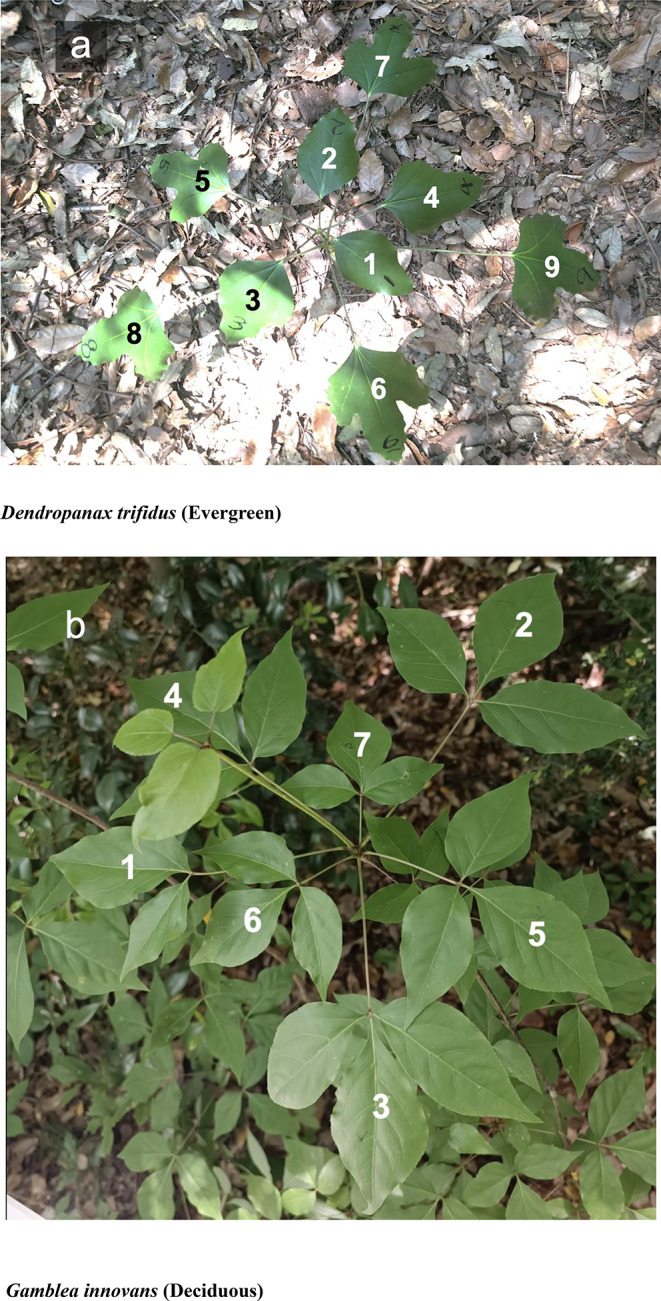
Distribution of leaves on a monoaxial stem of *Dendropanax trifidus* (**a**) and *Gamblea innovans* (**b**). The pictures were taken from the zenith. Numbers on each leaf indicate leaf order from the top in the cluster (The top leaf = 1, the bottom leaf = the largest figure)

Regarding the progressive change in petiole length and deflection angle with increase in node order for *D. trifidus* and *F. japonica*, the uppermost leaf blade was located closest to the stem, whereas the lowermost leaf blade was located the furthest from the stem (Fig. 5a). In contrast, for *G. innovans* and *C. sciadophylloides*, the topmost leaf blade was positioned furthest from the stem, whereas the lowermost leaf blade was located closest to the stem (Fig. 5b).

### Between-leaf-cluster comparisons of petiole length and deflection angle

**Fig. 6:**
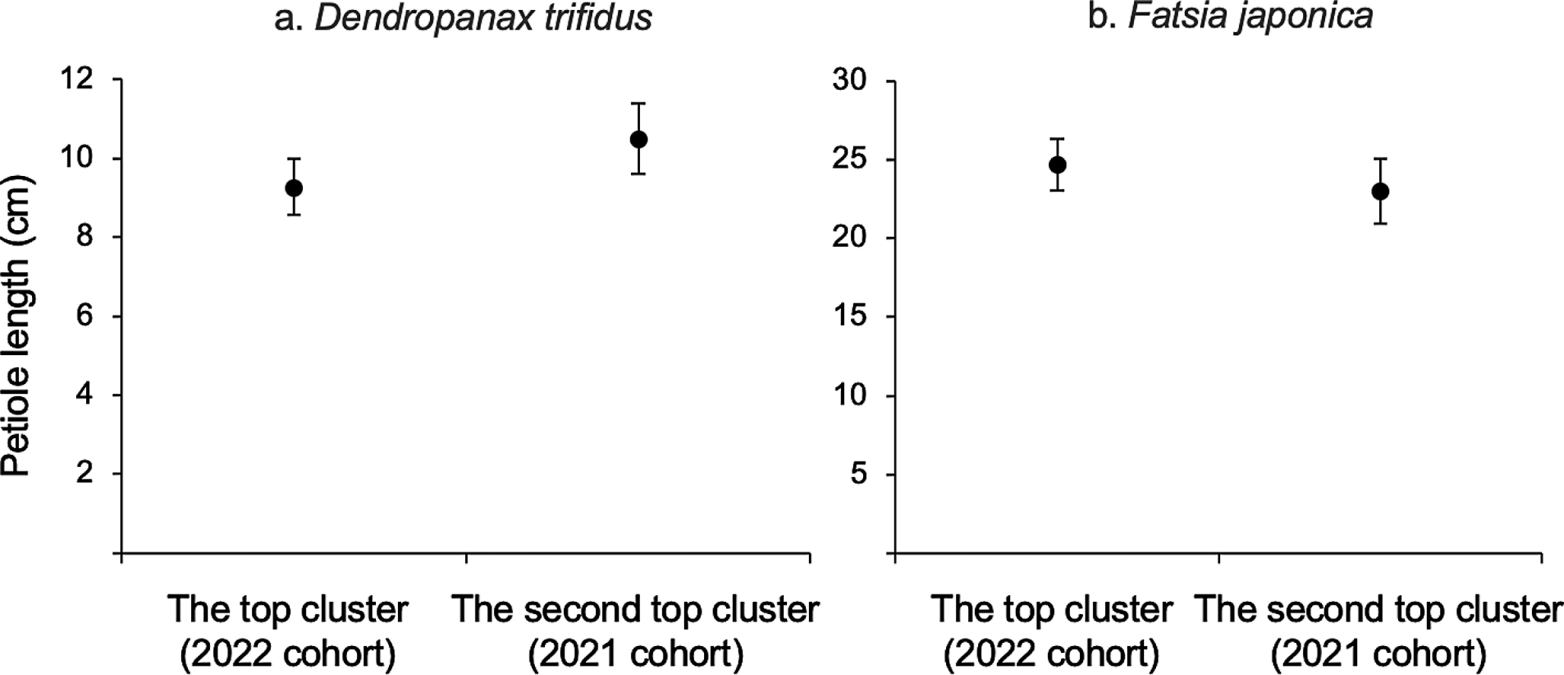
Comparison of mean petiole length between the first (top) and second leaf clusters for *Dendropanax trifidus* (*n =* 10) (**a**) and *Fatsia japonica* (*n =* 10) (**b**). Error bars indicate the standard error for each leaf cluster. No significant differences between the first and second clusters were observed for *D. trifidus* (paired-samples *t-*test, *p* = 0.10) and *F. japonica* (paired-samples *t*-test, *p* = 0.12)

The mean petiole length in the topmost cluster on the stem (= 2022 cohorts) was 9.3 cm for *D. trifidus* (standard error, SE = 0.7 cm) and 24.7 cm for *F*. *japonica* (SE = 1.7 cm), and that for the second cluster (= 2021 cohorts) was 10.5 cm for *D. trifidus* (SE = 0.9 cm) and 23.0 cm for *F*. *japonica* (SE = 2.1 cm). The mean petiole length did not differ signficantly between the topmost and the second leaf clusters in *D. trifidus* (paired-samples *t*-test, *p* = 0.10; Fig. 6a) and *F*. *japonica* (paired-samples *t*-test, *p* = 0.12; Fig. 6b).

**Fig. 7:**
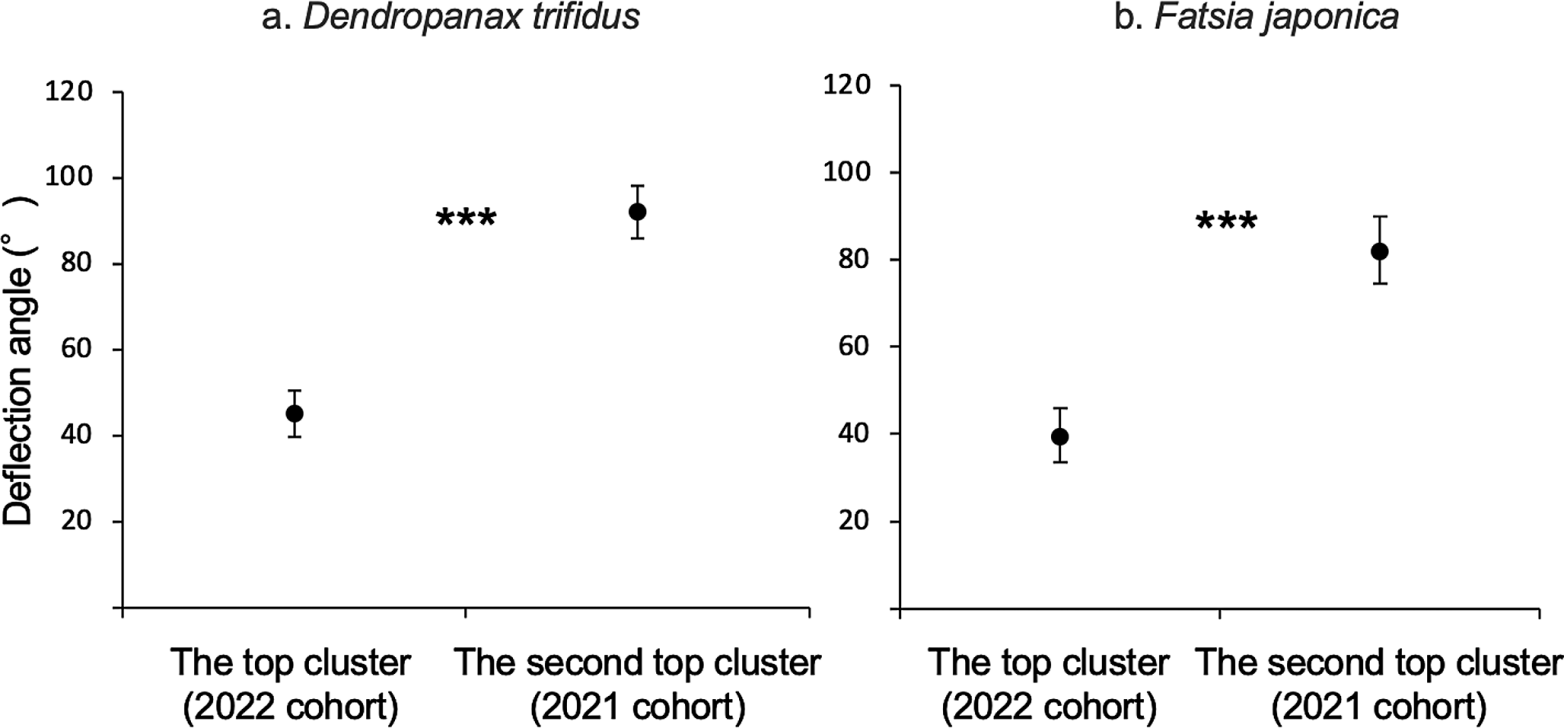
Comparison of mean deflection angle between the first (top) and second leaf clusters for *Dendropanax trifidus* (*n =* 10) (**a**) and *Fatsia japonica* (*n =* 10) (**b**). Error bars indicate the standard error for each leaf cluster. *** *p* < 0.001 (paired-samples *t*-test)

The deflection angle was compared between the topmost and the second leaf clusters in *D. trifidus* (Fig. 7a) and *F. japonica* (Fig. 7b). The mean deflection angle for *D. trifidus* and *F*. *japonica* was 45.0° (SE = 5.4°) and 39.6° (SE = 6.2°), respectively, in the topmost cluster (= 2022 cohorts), and 92.0° (SE = 6.2°) and 82.2° (SE = 7.6°), respectively, in the second cluster (= 2021 cohorts). The mean deflection angle of the topmost cluster was significantly smaller than that of the second cluster in both *D. trifidus* and *F*. *japonica* (paired-samples *t*-test, *p* < 0.001).

## Discussion

### Adaptive leaf arrangement to reduce self-shading within the topmost cluster

In a monoaxial tree, the progressive elongation of the petiole from the uppermost to the lowermost leaves has been considered to be a morphological adaptation to avoid self-shading within a leaf cluster (Takahashi and Mikami 2008; Yamada and Suzuki 1996; Yamada et al. 2000). Similarly, the progressive increase in deflection angle has been reported to act to reduce self-shading (Niklas 1988; Yamada and Suzuki 1996; Yamada et al. 2000). With these petiole length and deflection angle arrangements, the crown of *Adenocaulon bicolor* in Asteraceae which placed leaves horizontally in the understorey was greatly efficient in receiving light striking from zenith and optimized to minimise self-shading (Pearcy and Yang 1998). Saplings of *D. trifidus* and *F. japonica* conformed to those petiole length and deflection angle arrangements (Figs. 2 and 4), and the leaf arrangement might be adjusted for avoidance of self-shading.

In contrast, saplings of *G. innovans* and *C. sciadophylloides* had a different pattern of leaf arrangement. These species showed a progressive reduction in petiole length from the uppermost to the lowermost leaves in a leaf cluster (Fig. 2), which is opposite to the aforementioned pattern (Takahashi and Mikami 2008; Yamada and Suzuki 1996; Yamada et al. 2000). This pattern of change in petiole length was found in saplings of *C. sciadophylloides* in Hokkaido, Japan (Seino 2001). In addition, the saplings of *G. innovans* and *C. sciadophylloides* adjusted the leaf deflection angle in a unique manner. The uppermost leaves had the largest deflection angle and the angle declined with increase in node order (Fig. 4). While the importance of adjustment in the deflection angle has been emphasised for efficient light reception (Kikuzawa et al. 1996; Niklas 1988; Yamada et al. 2000), this pattern has not been reported previously.

Regarding the changes in petiole length and deflection angle, all four species realised a monolayered crown in which the leaves were arranged in one layer at a given height. This crown shape is known to provide an ideal leaf arrangement for optimal photosynthesis under a closed canopy (Givnish 1978; Horn 1971). For instance, Horn (1971) explained that a monolayered crown is one optimal form for photosynthesis in a light-limited environment where most light penetrates from the zenith. Givnish (1987) pointed out that a monolayered crown has the benefit of maximising reception of light by minimising self-shading within a crown, which was quantitatively verified by Pearcy and Yang (1998). However, the above-mentioned discussion is true only when most light penetrates from the zenith. Typically, this happens at noon in summer. But light environments in the understory are dynamic and change from morning to evening and with season (Valladares and Niinemets 2007; Yamada et al. 2014). We need to evaluate the efficiency of light reception under a dynamic light environment, too.

**Fig. 8:**
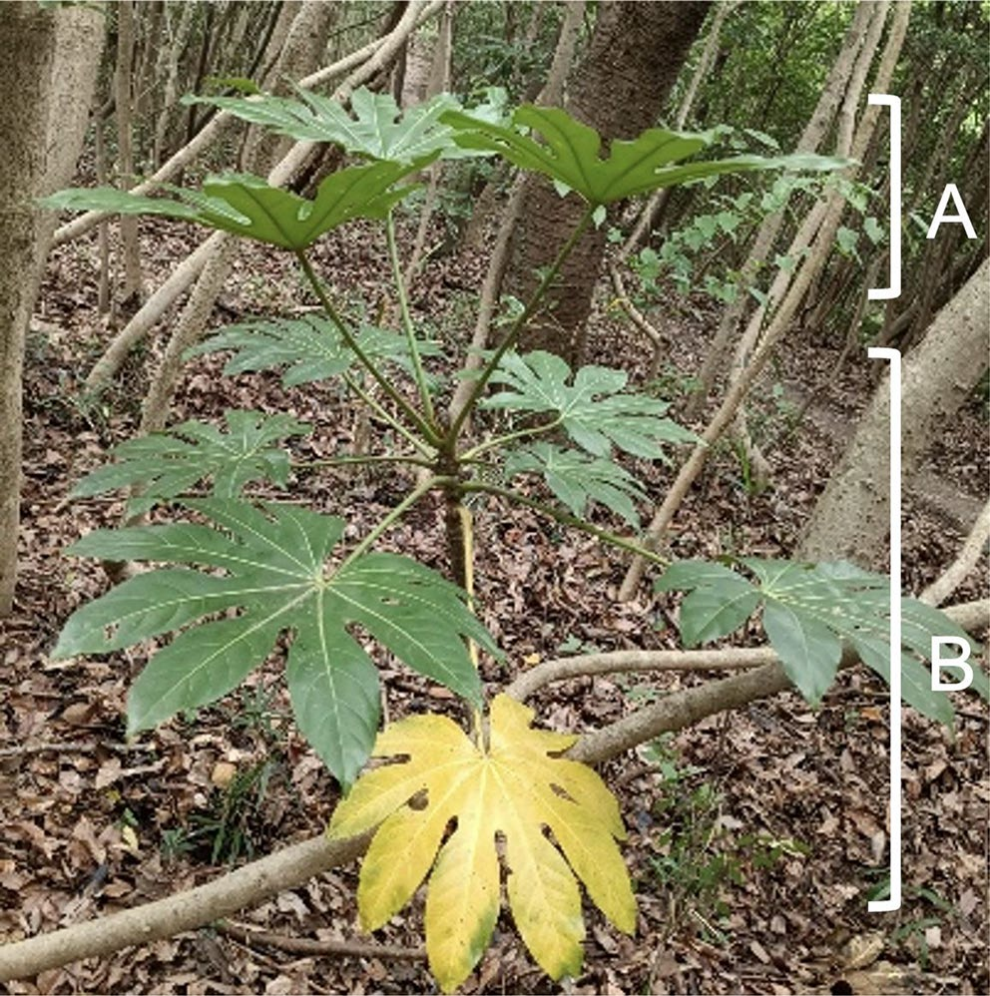
Adjustment of deflection angle on a monoaxial stem of *Fatsia japonica* to minimize the self-shading between the leaf clusters. The upper three leaves are the top leaf cluster (A; 2022 cohort) and the other bottom leaves are the second top leaf cluster (B; 2021 cohort)

In addition to adjustment of petiole length and deflection angle, a phyllotaxis of leaves plays an important role in reducing self-shading within a cluster (Gálvez and Pearcy 2003). Generally, leaf blades that are placed close to each other (Falster and Westoby 2003; Valladares and Niinemets 2007) or are placed close to the stem reduce light reception by increasing self-shading (Falster and Westoby 2003). The phyllotaxis adopted by the four species examined in this study (field observations, Figs. 5 and 8) were approximately all 3/15. This phyllotaxis is known to maximise the distance between adjacent leaves in node order in the horizontal plane (Gálvez and Pearcy 2003), suggesting that the species studied may have a high light reception efficiency (Niklas 1988) and may reduce self-shading. We need to test this idea in the future.

### Adaptive leaf arrangement to reduce self-shading between leaf clusters

The consequences of the leaf arrangements were identical among the four species, in leading to a monolayered crown and reduced self-shading, but the leaf arrangement strategy of the species pair of *D. trifidus* and *F. japonica* was entirely opposite to that of *G. innovans* and *C. sciadophylloides.* Thus, the question arises as to the functional difference between the two leaf arrangements.

A clue to answer this question may be in the relative leaf longevities of the species. *Dendropanax trifidus* and *F. japonica* are evergreen species that retain leaves for 3 years or more. Therefore, three or more leaf clusters are always present on a single stem. On the stem of these species, self-shading of leaves in a cluster by the leaves in an overlying cluster in addition to self-shading within a cluster must be problematic and requires adjustment of the deflection angle. However, *G. innovans* and *C. sciadophylloides* are deciduous species that completely turnover their leaves annually and retain only one leaf cluster on a stem. Therefore, for these species, self-shading between leaf clusters cannot occur and only self-shading within a cluster occurs.

Deflection angle variation is among the least expensive ways to improve leaf display (Poorter and Werger 1999). Comparison of the deflection angle between the leaf clusters suggested that *D. trifidus* and *F. japonica* showed a dynamic change in the deflection angle with time. The dynamic change in deflection angle plays a significant role in reducing self-shading between leaf clusters by two means. Firstly, leaves in the uppermost cluster of *D. trifidus* and *F. japonica* had significantly higher deflection angles than those in the second cluster. The difference in deflection angle resulted in greater vertical space between the first and second leaf clusters (Fig. 8). The vertical distance between the upper and lower leaves is known to reduce self-shading of the lower leaf by the upper leaf. For example, Takenaka (1994) mathematically studied shading of leaves in the lower leaf layer by leaves in the upper layer and demonstrated that increase in distance between the layers contributes to reduction in shading of the lower leaves. This observation suggests that increase in the space between the leaf clusters by adjustment of the deflection angle contributes to reduction in the degree of self-shading within a crown in an inexpensive way.

Secondly, increased deflection angles allow the leaves to be placed at a greater distance from the stem compared with those with a smaller deflection angle in a new leaf cluster. Consequently, some leaves in the old cluster can be deployed outside the crown projection of the new leaf cluster.

In evergreen species, increase in the deflection angle started when new leaves were flushed. To change the deflection angle over time, a leaf arrangement involving progressive increase in petiole length and deflection angle from the uppermost to the lowermost leaf is ideal because this leaf arrangement allows the species to adjust petioles without entangling adjacent leaves. For species in which the leaf arrangement exhibits progressive reduction in petiole length and/or in deflection angle, the deflection angle cannot be increased without the tangling of leaves during leaf movement. We conclude that an evergreen species is required to adopt a leaf arrangement with progressive increase in petiole length and deflection angle owing to this constraint. However, deciduous species are not subject to this constraint and thus have greater flexibility, and they can adopt a leaf arrangement incorporating progressive reduction in petiole length and deflection angle.

An additional question to address is: what benefits are offered by the leaf arrangement involving progressive reduction in petiole length and deflection angle over the leaf arrangement with progressive increase in petiole length and deflection angle? One possible explanation could be linked to the fact that the mass of leaf (and therefore the construction costs) increases progressively within a leaf cluster in deciduous species. The first leaf to emerge, which is also the smallest (and therefore cheapest), will support the growth of the next leaves, which will be larger and therefore more expensive. In most woody species, the annual elongation of the stem results in a gradual increase in internode length and leaf size. To adjust the deflection angle of the petiole without entangling adjacent leaves, evergreen species are required to develop the largest leaf first, which is an original and less common strategy for developing the stem and leaves. Needless to say, further studies are clearly needed to explore this.

Costs to construct a crown structure may be a clue to answer this question. Monoaxial trees reduce self-shading within a leaf cluster by placing leaves at a distance from the stem by making a long petiole (Poorter and Werger 1999; Yamada and Suzuki 1996). Because the petiole is non-assimilative, increased costs to petioles to reduce self-shading may limit the amount of the assimilative parts (leaf blades) in a crown, resulting in a decline in crown-level productivity. Besides, the cost investment on stem elongation for the increase in internode length is known to enhance light reception efficiency within a crown (Niklas 1988; Pearcy et al. 2005; Takenaka 1994; Yamada and Suzuki 1996). This is because the internode length between leaves on the stem increases vertical distance between the leaves, which contributes to reduce self-shading of the lower leaf by the upper leaf as mentioned above (Niklas 1988; Seino 2001). However, at the same time, it reduces biomass allocation on the assimilative parts (Pearcy et al. 2005). Therefore, the effectiveness of a crown structure to reduce self-shading should be evaluated from the viewpoint of construction costs, too. Possibly, the construction costs for the crown may differ between the different leaf arrangement patterns. Allometric relationships among petiole, leaf blade and stem mass should be compared among species.

### Reconsideration of the significance of monoaxial growth

Monoaxial growth, which does not produce any branches and deploys leaves directly on a single stem, has been discussed as a specialisation for rapid stem elongation (i.e., vertical stem growth) (Givnish 1978; Yamada et al. 2005). Petioles are more economically effective than branches because the majority of the mechanical strength of petioles is derived from turgor pressure and energetically inexpensive fibrous tissues (Givnish 1978). In addition, the present results suggest that a monoaxial tree can easily develop a monolayered crown suitable for growth in the understorey by adjusting the petiole length and deflection angle of the leaves. Therefore, monoaxial growth allows for both rapid growth in sunlight and survival in the light-limited understorey by creating a monolayered crown.

The adaptive significance of tree form for trees in the understorey has been discussed from the viewpoints of static functioning (which optimises the leaf display under the prevailing light condition) and dynamic functioning (which enhances the opportunistic use of light following disturbance) (Aiba and Kohyama 1996; Detto et al. 2022; Iida et al. 2011, 2012, 2014; King 1990; Kohyama 1987, 1992, 1993; Kohyama et al. 2003; Küppers 1989; Rahman et al. 2013; Thomas 1996; Thomas and Bazzaz 1999). Kohyama (1987) showed that the variation in tree form of understorey saplings was associated with the trade-off between horizontal growth for effective photosynthesis in the present (static functioning) and height growth for exploiting opportunities to reach a brighter light environment in the future (dynamic functioning). King (1990) found that the tree form of understorey saplings of canopy trees was better adapted for the dynamic functioning of height growth, whereas the permanent understorey trees exhibited a tree form that was better adapted for static functioning.

Saplings of the subcanopy tree species *D. trifidus*, *G. innovans*, and *C. sciadophylloides* may place great importance on the dynamic functioning of tree form to enhance vertical stem elongation because they must reach the subcanopy layer to be a reproductive mature tree. These species may realise faster vertical growth by greater energy investment in stem elongation to reach a well-lit environment. In contrast, *F. japonica* may show comparatively little vertical stem growth because stem elongation is not crucial for a permanent understorey tree. This species may invest energy predominantly in leaves as an assimilation organ to maximise photosynthesis. To verify this idea, measurement of the allometric patterns and vertical growth rates in relation to light environments of the four species studied herein is needed.
